# Prognostic Factors and Models for Predicting Work Absence in Adults with Musculoskeletal Conditions Consulting a Healthcare Practitioner: A Systematic Review

**DOI:** 10.1007/s10926-024-10205-y

**Published:** 2024-05-16

**Authors:** Gwenllian Wynne-Jones, Elaine Wainwright, Nicola Goodson, Joanne L. Jordan, Amardeep Legha, Millie Parchment, Ross Wilkie, George Peat

**Affiliations:** 1 Faculty of Medicine and Health Sciences, and Versus Arthritis/Medical Research Council Centre for Musculoskeletal Health and Work, School of Medicine, Keele University, Keele, ST5 5BG UK; 2 Aberdeen Centre for Arthritis and Musculoskeletal Health (Epidemiology Group), School of Medicine, Medical Sciences and Nutrition, Versus Arthritis/Medical Research Council Centre for Musculoskeletal Health and Work, University of Aberdeen, Aberdeen, UK; 3 Centre for Pain Research, University of Bath, Bath, UK; 4 Department of Rheumatology, Versus Arthritis/Medical Research Council Centre for Musculoskeletal Health and Work, Liverpool University Hospitals, Liverpool, L9 7AL UK; 5 NIHR Applied Research Collaboration for Greater Manchester, University of Manchester, Manchester, M13 9PL UK; 6 Bath Centre for Pain Research, University of Bath, Bath, UK; 7 Centre for Applied Health & Social Care Research (CARe), Sheffield Hallam University, Sheffield, UK

**Keywords:** Systematic review, Absence, Work absence, Prognosis, Musculoskeletal pain

## Abstract

**Purpose:**

It is difficult to predict which employees, in particular those with musculoskeletal pain, will return to work quickly without additional vocational advice and support, which employees will require this support and what levels of support are most appropriate. Consequently, there is no way of ensuring the right individuals are directed towards the right services to support their occupational health needs. The aim of this review will be to identify prognostic factors for duration of work absence in those already absent and examine the utility of prognostic models for work absence.

**Methods:**

Eight databases were search using a combination of subject headings and key words focusing on work absence, musculoskeletal pain and prognosis. Two authors independently assessed the eligibility of studies, extracted data from all eligible studies and assessed risk of bias using the QUIPS or PROBAST tools, an adapted GRADE was used to assess the strength of the evidence.

To make sense of the data prognostic variables were grouped according to categories from the Disability Prevention Framework and the SWiM framework was utilised to synthesise findings.

**Results:**

A total of 23 studies were included in the review, including 13 prognostic models and a total of 110 individual prognostic factors. Overall, the evidence for all prognostic factors was weak, although there was some evidence that older age and better recovery expectations were protective of future absence and that previous absence was likely to predict future absences. There was weak evidence for any of the prognostic models in determining future sickness absence.

**Conclusion:**

Analysis was difficult due to the wide range of measures of both prognostic factors and outcome and the differing timescales for follow-up. Future research should ensure that consistent measures are employed and where possible these should be in-line with those suggested by Ravinskaya et al. (2023).

**Supplementary Information:**

The online version contains supplementary material available at 10.1007/s10926-024-10205-y.

## Introduction

Sickness absence remains a significant cost to developed countries accounting for between 0.5 and 2% of lost GDP in European countries alone [1]. There is evidence that the majority of employees taking a period of absence will only take a short time away from the workplace [2]. However, across all working ages there is a small minority that go on to take longer-term absence, variously defined as greater than 4 weeks absence, greater than 6 weeks and up to 3 months [3–5]. However long-term absence is defined, it is the small proportion of people going on to long-term absence who make up the majority of the costs associated with absence from the workplace [6].

At present, it is difficult to predict which employees, in particular those with musculoskeletal pain, will return to work quickly without additional vocational advice and support, which employees will require this support and what levels of support are most appropriate. Consequently, there is no way of ensuring the right individuals are directed towards the right services to support their occupational health needs. There is a growing evidence base around the usefulness of stratified care approaches to delivering healthcare, whereby prognostic information is used to allocate individuals to sub-groups with matched recommended treatments or interventions [7, 8], stratified care has also been demonstrated to be a cost effective model [9, 10]. This approach has not been developed in occupational health yet, but the principles behind it could be used to ensure that scarce occupational health resources are targeted towards those individuals who need more support, whilst also providing reassurance to those for whom sickness absence is unlikely to be become longer term. To allow a stratified care approach to be developed it is important to identify which factors predict work absence and to examine the utility of current prognostic models or tools [11].

It is anticipated that prognostic factors for work absence will be varied. Sickness absence is a complex concept, influenced not only by an individual’s health (or severity of health condition), but also by psychosocial variables, macro system variables (e.g. health services and workplace systems) and wider societal systems (e.g. sickness benefits policies) [12]. For many individuals, decisions about sickness absence will be made in the context of their own health, their own workplace and their own attitudes and beliefs. To support the management of the variety of prognostic variables anticipated in this systematic review, it may be possible to identify some common “core” concepts that can be used to predict the likelihood that individuals will go on to longer-term absence. These concepts can be organised around a framework, such as the disability prevention framework [13], which structures the impacts on the health and work relationship into the “core” concepts of personal systems, healthcare systems, workplace systems and compensation system. Within each of these core concepts are sub-groups which allow an examination of the potential predictors of work absence on a more granular level.

Whilst there is a body of literature examining predictors of sickness absence [14–16], there have been no systematic reviews that comprehensively consider which factors are predictive of work absence or the usefulness of prognostic models or measurement tools in identifying those who will have longer-term work absence. Furthermore, there is no evidence focussed on the prediction of absence duration in those that are already absent from work and presenting to primary care. This is a key timepoint in which to be able to provide evidence-based advice and guidance or to refer patients to appropriate services to support them with their health and work in particular those with long-terms conditions, such as musculoskeletal pain (NICE 2019). Therefore, the primary aim of this review will be to identify prognostic factors for duration of work absence in those already absent and examine the utility of prognostic models for work absence.

## Methods

This systematic review is reported using the PRISMA guidance [17] and the recommendations of Riley et al. [18] for undertaking systematic review of prognostic factors. The review was prospectively registered with PROSPERO (CRD42020219452).

### Search Strategy

An experienced information specialist designed and conducted the searches using a combination of subject headings and key text words. The full search strategy is reported in the online supplement. The following eight databases were searched from their inception to 6th October 2020: MEDLINE; EMBASE; CINAHL; AMED; PsycINFO; HMIC; Business Source Complete; Cochrane Library (CENTRAL), a full updated search was run on 18th September 2023.

### Inclusion and Exclusion Criteria

#### Participants/Population

Studies including employed adults who were on sick leave and seeking or receiving healthcare for a musculoskeletal condition were included. If studies reported on participants who were unemployed, not on sick leave or working modified or alternative duties they were excluded. Studies where participants did not have musculoskeletal conditions or where these were as a result of acute trauma or injuries (such as fractures) were excluded as were studies where the participants had inflammatory arthritis or surgical intervention for their condition.

#### Study Setting

Studies set in primary (first contact) care, community care and workplace settings where employees have sought healthcare have been included. Studies conducted in hospital populations, emergency care, tertiary care, or rehabilitation centres were excluded.

#### Study Type

Cohort studies (prospective and retrospective) with an integrated health and work focus were included. Additionally, prognosis studies based on randomised controlled trial data and/or case–control studies were included alongside those papers that reported on tools or models used to predict work absence and summarise the predictive performance of the tool or model used. All other study designs were excluded.

### Prognostic Factors

The predictive performance of all identified prognostic factors or prognostic models were evaluated. We did not limit the factors that could be included allowing a full exploration of the breadth of prognostic factors examined in the literature.

### Outcomes

The outcome of interest for this review was work absence. Prognostic factors for RTW will be reported in a separate publication. Work absence could be measured in any way (e.g. self-report, employer records, or insurance records) and at any follow-up time point. Definitions of absence were extracted from the studies to allow a comparison of outcome measures.

The strength of association of individual prognostic factors with the outcome were extracted from studies. Where the outcome was binary (absence from work yes versus no) the odds ratio, relative risk, or time to event data were extracted, where the outcome was continuous (e.g. number of days absent from work) the mean differences were extracted.

### Screening and Data Extraction

All screening and data extraction was undertaken by pairs of review authors independently. Any disagreements were resolved through consensus bringing in a third reviewer if necessary. The screening of titles and abstracts was undertaken using Rayaan software and the full text screening and data extraction undertaken using Covidence software.

A standardised data extraction form was developed and tested using MS Excel before being used to extract data from the included studies. Study-level data were collected on study design (primary care, community, population/national based, health records (primary care), health records (secondary care), health records (insurance), occupational health, outpatients, hospital/rehabilitation, other secondary care and other setting (defined)). Data were also collected on inclusion criteria, population description, definition of outcome, outcome data type (binary, continuous, time to event), follow-up time period, prognostic factor and description, variables used in adjustment of the analyses, adjusted and unadjusted estimates of the association between the prognostic factor and the outcome. Where studies reported on a model, measures of the model’s performance were also extracted.

### Quality Assessment

The Quality In Prognosis Studies (QUIPS) tool [19] was used to assess potential bias in prognostic factor studies and Prediction model Risk Of Bias ASsessment Tool (PROBAST) [20] for studies reporting prognostic models. Two authors independently assessed risk of bias for each study reported as unclear, high, or low risk of bias, for each domain of the tools (QUIPS and PROBAST). These were compared between each pair and any disagreements resolved through discussion or by consulting a third reviewer if necessary.

### Assessment of the Strength of Evidence

GRADE was used to assess the strength of the evidence. This method takes into account a number of factors allowing a judgement to be made on the body of evidence overall rather than focusing on individual studies as with risk of bias.

For each of the groups of prognostic factors reported below, GRADE was used to assess the risk of bias with evidence downgraded where more than half of studies had moderate or high risk of bias. Additionally, evidence was downgraded where there was inconsistency in estimates of effect and/or heterogeneity between studies in the definition of the prognostic factor. Downgrading was also applied if there was any indirectness defined as follows: not all the participants were absent and separate results were not reported for those that were; only a subset of the population was represented (e.g. just males/females); the prognostic factor was not fully represented e.g. only a subset of those reporting absence were included. Finally, evidence was downgraded if there was any imprecision which included fewer than 2 studies in each prognostic factor grouping or if there was an insufficient sample size to detect a difference for the prognostic factor in most of the studies.

When considering the strength of the evidence around the prognostic models predicting absence from work an adapted GRADE was used. This was primarily to ensure that appropriate consideration of the performance of the included models was included, the guidance from Foroutan et al. [21] was used. Evidence was downgraded where calibration was imprecise with wide variation in point estimates overall and wide confidence intervals.

Evidence was deemed to be high quality if none of the domains were downgraded, moderate quality if one of the domains was downgraded, low quality if two were downgraded and very low quality if three or four were downgraded.

### Data Synthesis

A narrative synthesis was planned to allow for variation in outcome measures, settings and prognostic factors included in the studies within this review. Whilst the Popay narrative synthesis framework [22] had been planned to be used, the more recent Synthesis Without Meta-analysis (SWiM) framework was used to structure the data synthesis [23], this framework provides a guide with which to group, describe and report the results of this systematic review and was considered a more appropriate approach to synthesising the evidence in this review.

#### Grouping of Prognostic Factors

**Fig. 1:**
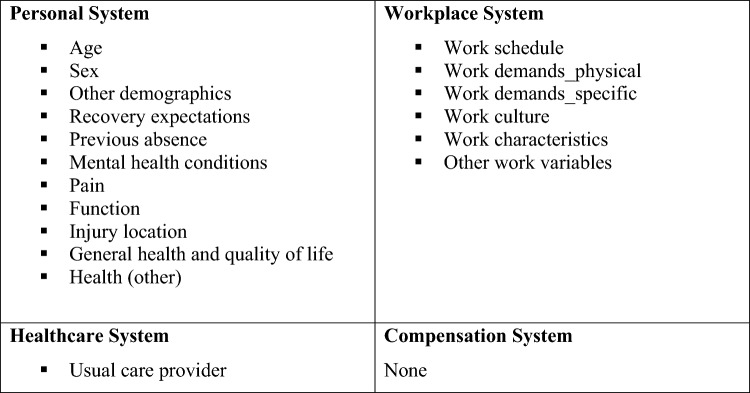
Overarching groups and categories of prognostic factors

Due to the wide variation in prognostic factors measured within the studies in this review, they were grouped into broad domains. In total there were 110 individual factors identified which were grouped via discussion within the team into 17 broad categories. These categories were further grouped for synthesis to broadly fit the categories of the Disability Prevention Framework [13]; however, there were no variables that could be grouped into the compensation system concepts and just one variable reporting a healthcare prognostic factor (Fig. 1).

#### Description of Standardised Metric

This paper aimed to identify prognostic factors for work absence and therefore, a range of metrics were extracted and recorded. For binary outcomes, odds ratios (OR), relative risk (RR) and risk reduction were recorded, for continuous outcomes mean differences were recorded and for time to event outcomes hazard ratios (HR) were recorded. Where available the adjusted and unadjusted effect estimates were recorded. These metrics for reporting prognostic factors are recommended in the CHARMS-PF checklist [24, 25].

#### Methods of Synthesis

There was significant inconsistency across prognostic factors in terms of measurement and analysis and also inconsistency in outcome measure so a formal meta-analysis was not possible. However, data were sufficient to report the range and distribution of observed effects as well as identifying whether there was evidence of an effect in one or more studies examining the same prognostic factor and also to explore the direction of any effects seen.

## Results

**Fig. 2:**
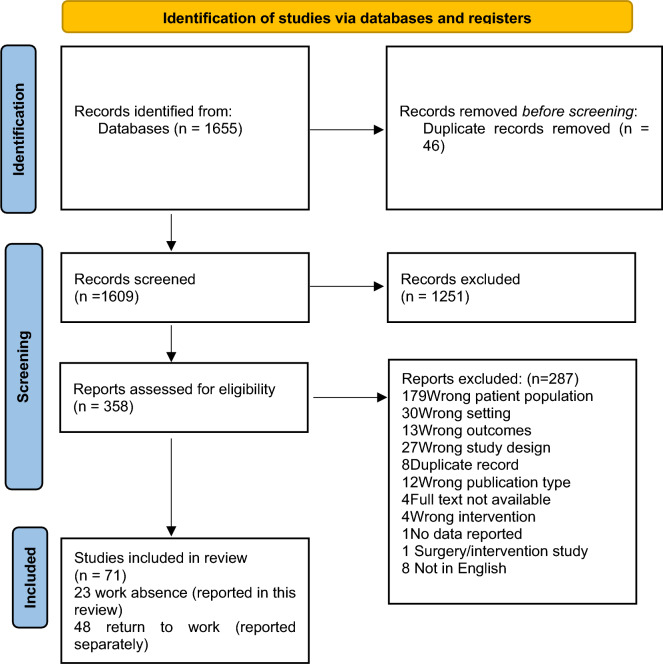
Flow diagram of study selection. From Page et al. [17]. For more information, visit: http://www.prisma-statement.org/

The searches returned 1655 references. Following de-duplication 1609 references remained. After completing screening of titles and abstracts, 358 full texts were retrieved for assessment of eligibility with 23 studies included in the current systematic review and a further 48 studies identified for inclusion in a separate review reporting RTW as the outcome (Fig. 2).

### Results: Prognostic Factors

Across all 23 studies 111 individual prognostic factors were identified. There was considerable inconsistency in study design, prognostic factor measurement, outcome measurement, time point of follow-up and analysis methods across the included studies meaning a meta-analysis was not appropriate. Prognostic factors were grouped into 18 themes for ease of management and reporting (Fig. 1) which can be considered within three domains of the disability prevention framework: personal system, workplace system and healthcare system.

#### Description of Included Studies: Prognostic Factors

Table 1 provides an overview of the included studies. In summary, the studies were mainly conducted in North America (9 in Canada and 4 in the USA), this was followed by the Netherlands with 3 studies, 3 originating in Australia, with the rest from other European countries (5 studies).

**Table 1:** Study characteristics (prognostic factors)

Paper ID	Recruitment dates	Country	Participants	Study setting	Total number of participants	Mean age (years)	Absence definition	Absence duration (baseline)	Follow-up (years)	Prognostic factors measured
Abasolo et al. [39]	1998–2001	Spain	Patients receiving temporary work disability	Primary care	*n* = 3311 Women: 1656 (50%)	41 (±12)	Days of sick leave per episode	Median 13 days	2	Age Female sex Self-employed Married Low educational level Responsible for most or all of the household chores Work position covered Unemployed Manual worker Seated for long periods Must adopt squatting position Must stand up for long periods Physically demanding job Must perform anterior flexion of the neck
Abasolo et al. [39]							Recurrence of temporary work disability—any successive temporary work disability during the study period			Female sex General work regimen Married Low educational level Responsible for most or all of the household chores Work position covered Unemployed Manual worker Seated for long periods Must adopt squatting position Must kneel frequently Must stand up for long periods Physically demanding job Must perform anterior flexion of the neck Must perform anterior flexion of the trunk Must perform later flexion of the trunk Duration of previous temporary disability episode (per day)
Abenhaim et al. [26]	1988	Canada	Aged 15–65 with at least 1 day of compensated absence from work, injury to the thoracic, lumbar, or sacral segments of the spine	Medical/healthcare records	*n* = 1848 Women: 425 (23%)	Not reported	Compensated absence Chronicity defined as accumulation of 180 days or more of compensated absence from work over all episodes that occurred during the study period	At least 1 day	2	Diagnosis (specific versus non-specific)
Gabel et al. [31]	Not reported	Australia	Acute MSK injury to the spine, upper or lower limb sustain at work within the previous 5 weeks	Outpatients	143 Women: 61 (43%)	Mean 38.9 (SD 10.5); range 18–65	Long-term absence defined as > 28 paid days off No absenteeism 0 paid days off	72% absent for 1–28 days 26% absent for > 28 days	0.5	21-item Orebro Musculoskeletal Pain Questionnaire 12-item Orebro Musculoskeletal Pain Questionnaire
Sheehan et al. [43]	2010–2015	Australia	Low back pain claims with a minimum of 4 recorded primary care service payments greater than 2 weeks paid time loss and from the workers compensation schemes of 3 Australian states	Health records (insurance)	18,696 claims (not necessarily individual people) Women: 6916 (7%)	Not reported	Working time lost, defined as the number of weeks of income support payments paid (measured in paid calendar weeks)	At least 1 week	5	Continuity of care with usual care provider measured with the usual provider continuity index
Lederer et al. [32]	2000–2002	Canada	Claim incurred between 1st Jan 2001 and 31st Dec 2003, benefits granted for at least 90 days and coded as a new event (not a relapse) with an upper body injury site	Health records (insurance)	2210 Women: 9032 (40.9%)	Not reported	Time on compensated benefits calculated as the difference between the date of injury and date of the last payment of benefits for a maximum of a 3 year period	At least 3 months (90 days)	0.5	Age Gross annual income Dependents Area of residence Industry Injury type Injury site Claim history (previous 10 years)
Lotters et al. [34]	Not reported	The Netherlands	On sick leave due to non-specific musculoskeletal disorders for 2–6 weeks	Occupational health	253 Women: 76 (30%)	43 (SD9)	Duration of sickness absence	2–6 weeks	1	Perceived pain—Low back pain Perceived pain*—*other MSK Perceived physical workload Visiting a specialist 12 months prior to current sickness absence Own perception of RTW Presence of sciatica
Nordin et al. [29]	March 1994–July 1995	USA	All employees having a first episode of non-specific low back pain (defined with ICD9 codes) within 1 week of episode onset	Hospital/rehabilitation	162 Women: 33 (20%)	39.9 (range 20–69)	Number of days (ascertained through computerised company records)	1 week	0.12	Abnormal heel walk Oswestry quartile Work-related injury Exposure to whole body vibration Physically heavy work
*Okurowski et al. [28]	Jan 1997–Mar 2000	USA	Cases who were out of work at 3-month post-injury as a result of uncomplicated low back pain and who had similar level of nurse case manager evaluation	Health records (insurance)	986 Women: 256 (26%)	Not working: 37 (SE 0.41) Working (SE 0.54)	Working or not working at 6 months	3 months	Not reported	Age Timeliness of referral Language barriers Attorney involvement
Richter et al. [35]	Nov 2004–Dec 2006	The Netherlands	New work disability insurance claim episodes from those with non-specific MSK symptoms who were unable to fulfil their job for more than 25% according to medical assessment	Health records (insurance)	276 Women: 20 (7%)	45 (SD 7)	claim duration, defined as the number of calendar days the participant received work disability compensation between completion of the baseline questionnaire and one-year follow-up, without adjustment for the level of work disability (gross duration). The end of a claim period was defined as having less than 25% work disability according to a medical assessment, with a minimum duration of 4 weeks	Mixed duration but up to 6 months	1	Age Gender General health History of similar symptoms Pain severity previous 6 months Location of MSK symptoms (upper extremity; back; lower extremity; multiple locations Duration of symptoms (2–6 months; > 6 months) Functional status neck pain Functional status back pain Insured daily compensation Deferment period Fear of movement Self-predicted timing of RTW Job satisfaction Willingness to participate in RTW
Selander et al. [36]	June 2003–June 2004	Sweden	Participants were on long-term sick leave (over 4 months) due to back pain problems	Hospital/rehabilitation	347 Women: 160 (46%)	Mean 42 (male) Mean 41 (female)	Absence: Bivariate outcome: Successful rehabilitation if client had lower degree of sickness absence or none at all c.f. their baseline absence. Unsuccessful rehabilitation if client received same or more sickness allowance	Between 3 and 11 weeks Mean 63 days from date of injury	0.5 and 1	Age General health Vitality Internal locus of control
Shiels et al. [44]	Not reported	UK	Not reported	Primary care	864 Women: 411 (47.6%)	43.1	(1) total duration of sickness episode (calculated by totalling all periods of incapacity on the sickness certificates In the case of issue not being continuous, separate episodes were assumed); (2) greater than 28-week incapacity	Not reported	Not clear but greater than 0.1 years	Age Sex Deprivation Type of MSK disorder
Smith et al. [40]	1 Jan 2005–31 Dec 2007	Australia	Wage replacement claimants with an incapacity start date between 1st Jan 2005 and 31st Dec 2007 which were either (mental health^†^), back or upper extremity claims from full- or part-time employees	Health records (Insurance)	10,899 Women: 4197 (38.5%)	15–24y = 6.2%; 25–34y = 17.7%; 35–44y = 29.7%; 45–54y = 36.8%; 55+ = 16.9%	No. of days of total wage replacement over 2 years from first day of absence	1 day	2	Age when injured Gender Prior claim Days between injury date and first day of compensation Employment type Occupational strength requirements Occupational time pressure Occupational autonomy Managing employer size Industry Year
Steenstra et al. [41]	1 Jan–30 Jun 2005	Canada	Compensated work absence after an uncomplicated back injury	Health records (Insurance)	1422 Women: 552 (38%)	41.3 (SD 10.5)	Time on compensation benefits until RTW and time to further period of compensated absence defined as recurrent of same injury	4 weeks	2	Age Gender Previous claim Physical demands manual Language (non-French or English) Union member Early RTW programme Employer continued salary Employer doubt about work related injury No recovery expected Worker signed RTW forms Public transport to work Functional abilities Opioid prescription
Truchon et al. [37]	Oct 2006–Nov 2008	Canada	Workers receiving income replacements benefits because of common low back pain. Aged 18 or over and affected by a first or new episode of low back pain in the last 12 months. On sick leave for a minimum of 28 days but no longer that 83 days	Population/National based	535 Women: 218 (40.7%)	42 (SD10)	Number of days of absence. Calculated on the basis of dates supplied by the participants about work events during the phone interviews at follow-ups (e.g. returns to work, recurrence of disabling LBP). A total absence period was calculated for each participant from injury date (minimum 35 days; maximum 340 days). This absence period could include multiple sick leaves. Periods of light duty work were considered as returned to work periods even if treatments were provided one or many days per week. Participants were divided into two groups on the basis of this absence period: 182 cumulative days and less and more than 182 cumulative days	Long term >/= 4 weeks	1	Fear avoidance beliefs work (FABQ-W) RTW expectations (time) Annual family income (pre-tax) Last level of education attained_elementary Work schedule irregularity Work concerns
Turner et al. [33]	Jul 2002–Jun 2003	USA	Workers how submitted workers compensation claims for work-related back pain and received at least 1 day of temporary total disability wage replacement (i.e. had at least 4 days of work disability as required for receiving wage replacement)	Health records (insurance)	1068 Women: 328 (31%)	39.2 (11.1)	Wage replacement compensation for temporary total disability (“work disability”) 6 months (180 days) after claim submission. Number of days of wage replacement receipt in this period was also examined (“work disability duration”) Temporary total disability payments are stopped when a worker returns to work or is judged to be medically stable and able to work	Mean 21.1 days (SD 9.7)	0.5	Recovery expectations Mental Health Catastrophising Blame (work) Blame (someone/something else) Relations with co-workers Work fear avoidance
Van Duijn et al. [38]	Not reported	The Netherlands	Participants were on sick leave with musculoskeletal complaints for between 2 and 6 weeks	Occupational health	262 enrolled data presented on 164 who completed follow-up data Women: proportion not clear	43 (9)	Duration of sick leave time until RTW on full duty RTW but in a modified work capacity (reduced hours, modified work during sick leave advised by OH)	2–6 weeks	1	Modified work Age Duration in job Prior sick leave Chronic health Severity of pain Disability Physical general health Quality of life
Westman et al. [42]	1998–2000	Sweden	Employed 18–65-year olds sick listed between > 28 and < 180 days and/or had consulted the doctor about the same problem at least three times in the last 12 months (as recorded by the referring physicians)	Primary care	158 Women: 110 (69%)	47 (range 24–65)	Worsening or improving sick leave during follow-up	Not clear	3	Orebro Musculoskeletal pain questionnaire score Function Pain Distress Fear avoidance RTW expectations Coping

*SE* standard error, *SD* standard deviation

*Study also presents a prognostic model

^†^Not included in the analyses

The majority of studies were conducted using records from healthcare insurance databases (10 studies) with 3 studies conducted in primary care, 4 in occupational health and the remaining 6 studies from other settings. Prospective cohort studies were the most common design (15 studies) with retrospective cohorts (*n* = 5) and health record reviews (*n* = 2) being less frequently employed.

The outcome measure of work absence was defined differently in all studies, although the number of days absence from work was the most commonly used metric, this was calculated differently across studies, from

- A simple count of days from company records as reported by Abenheim et al. [26] and Bosman et al. [27].
- Working or not working at 6 months as reported by Okurowski et al. [28].

- To more complex calculations such as that reported by Nordin et al. [29] where the days of absence were recorded from phone interviews with participants or where compensated days were calculated as reported by Abenheim et al. [26] and Lederer et al. [30].

The length of follow-up also varied ranging from 6 months or less in five studies [28, 29, 31–33], 12 months in five studies [34–38], 2 years in four studies [26, 39–41] and two with longer-term follow-up at 3 [42] and 5 years [43]. One study was not clear in the reporting of duration of absence, Shiels et al. [44], however, reported participants were followed up for greater than 1 month.

Most of the studies reported prognostic factors only; however, there were 13 prognostic models identified [27, 28, 33, 35, 37, 40–42, 45–49], some were models that had already been developed and were being tested in new populations and others were developed within a specific population.

#### Risk of Bias: Prognostic Factors

**Fig. 3:**
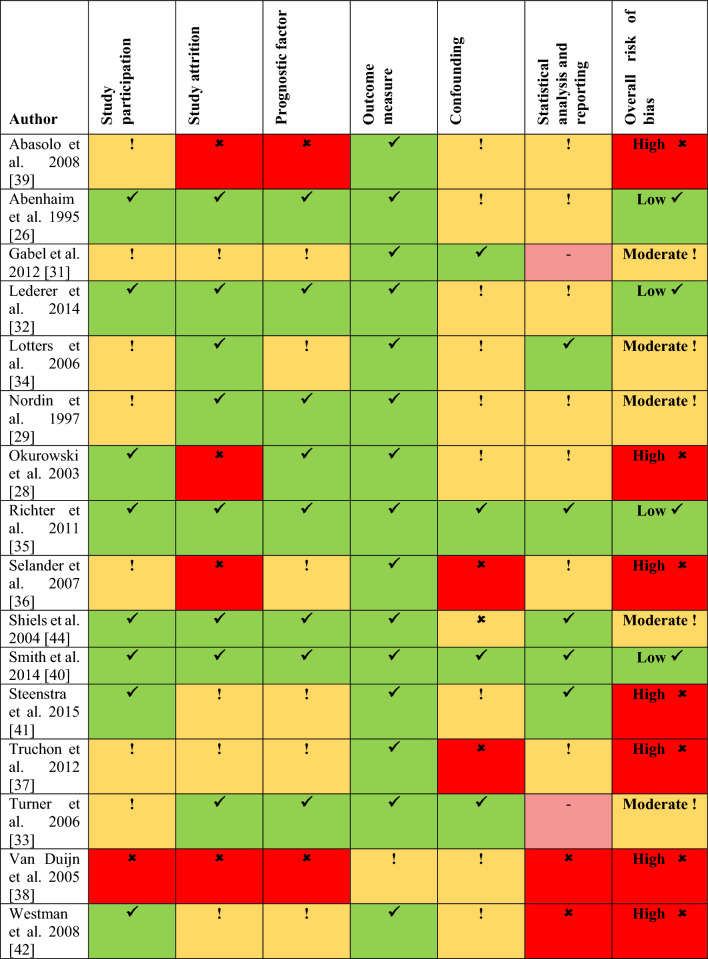
Risk of bias (QUIPS)—domain summary assessments

The summary judgements for each domain of the QUIPS tool are reported in Fig. 3. Six of the included studies had at least one domain that was considered high risk [28, 36–39, 42] and a further study was considered high risk overall due to the number of domains scoring moderate risk of bias [41]. The most common reason for a high risk of bias was study attrition, either through a large number of participants being lost to follow-up or studies not reporting attrition or the potential effect of this on the studies’ findings. The high risk of bias of these studies is reflected in the GRADE assessment. Just three studies were considered at low risk of bias [26, 35, 40], with the remaining five studies at moderate risk of bias with a lack of consideration or reporting of potential confounding being the most common domain to be reported as moderate risk.

#### Strength of the Evidence: Prognostic Factors

GRADE was used to assess the strength of the evidence and reported by grouping the prognostic factors into the 17 broad categories reported above (Fig. 1). For each of the categories the supporting research was assessed using an adapted GRADE criteria and an overall judgement was agreed (Table 3).

None of the categories were judged to be strong, with the strength of the evidence being low and with one theme “function” having a very low grading. The only factor that demonstrated an overall protective effect was age where an increasing age was associated with a lower risk of absence (reported in 9 studies) [28, 32, 35, 36, 38–41, 44]. All other themes were associated with a higher risk of absence; however, comparisons within and between themes were impeded by the differing measures used across the studies.

#### Summary of Findings by Personal Systems

Most of the categories included in personal systems (Fig. 1) reported on inconsistent measures and outcomes and therefore provided a very mixed picture in terms of the contribution of that category to predicting work absence. There were some specific categories that warrant a fuller reporting as the direction of effect tended towards a more consistent direction, these are age, sex, recovery expectations and previous work absence.

##### Age

Age was reported in many different ways across each of the studies; however, it did demonstrate an overall protective effect where increasing age was associated with a lower risk of absence. For example, Steenstra et al. [48] reported age in 10-year increments from 15 to 25 years through to 55–65 years and found that for time on benefits there was a dose-response effect when compared to the 25–35-year age group, with the 15–25-year age group reporting increased absence (hazard rate ratio 1.27; 95% CI 1.00, 1.60) and the older age groups reporting a lower risk of absence with increasing age.

##### Sex

Those studies reporting sex as a prognostic factor demonstrated no consistent direction of effect [32, 35, 39–41, 44, 49]. For example, Abasolo et al. [39] found that women were less likely to experience temporary work disability when compared to men (HR 0.84 95% CI 0.78, 0.90); however, there was no difference in recurring work disability (HR 1.13 95% CI 0.97, 1.32). Richter et al. [35] reported that men were more likely to experience absence at follow-up when compared to women although not statistically significantly (HR 1.59 95% CI 0.78, 1.22). Steenstra et al. [41] found that women were more likely to experience a recurrence of absence over two-year follow-up (hazard rate ratio 1.36 (95% CI 1.09, 1.70).

##### Recovery Expectations

Four of the studies included reported on recovery expectations, broadly the better a participants’ recovery expectations the better the outcome [33–35, 48]. For example, Turner et al. [33] reported a dose-response effect with recovery expectation of 0 (on a 0–10 scale) having an odds ratio of 9.18 (95% CI 5.00, 16.84) for 6-month work disability (defined as number of days on wage replacement) when compared to those with a very high recovery expectation. This odds ratio reduced to 1.95 (95% CI 1.18, 3.20) for those reporting a high recovery expectation of 8–9 (on a 0–10-point scale). However, it should be noted that there was no protective effect of recovery expectations in the study by Turner et al. People who were unable to identify when they would return to work had a poorer outcome in the study by Richter et al. [35] HR 0.23 (95% CI 0.15, 0.34) and those who reported they would return to work over a month later reported a HR of 0.24 (95% CI 0.15, 0.38) indicating that a poorer recovery expectation was associated with a reduced “risk” of getting back to work.

##### Previous Absence

Four studies reported on previous work absence and whether it can predict future absence [32, 38, 39, 41]. All studies measured previous work absence as a previous “claim,” the general direction of effect was of previous work absence being predictive of future work absence. For example, Lederer et al. [32] reported a HR of 0.91 (95% CI 0.87, 0.94) for previous claim history in the past 5 years (for return to work) and Van Dujin et al. [38] found that prior sick leave (in the past 12 months) had a HR of 1.50 (95% CI 1.03, 2.17) at univariate analysis, but this variable was not included in the multivariable analyses.

There was some evidence that mental health may contribute to absence with Turner et al. [33] reporting that mental health below the population mean (measured using the SF-36-v2) was associated with increasing absence; however, these were not significant results (< 2 standard deviations (SD) below the mean OR 1.59 (95% CI 0.82, 2.08), 1–2 SD below the mean OR 1.84 (95% CI 0.99, 3.42)  and < 1 SD below the mean OR 1.66 (95% CI 0.91, 3.03). Turner et al. found no effect on absence related to catastrophising or blame. A high fear of movement was not reported to be associated with absence by Richter et al. [35] OR 0.94 (95%CI 0.67, 1.33); however, Turner et al. [33] found that a high fear avoidance at 5–6 points measured with the Fear Avoidance Behaviour Questionnaire was associated with absence OR 4.64 (95% CI 1.57, 13.70) and at 3–5.9 points OR 2.96 (95%CI 0.98, 8.90).

General health and quality of life were reported by Richter et al. [35] (general assessed with one question good versus poor) and Selander et al. [36] (using the SF36) but there was no evidence of a relationship with work absence. Van Dujin et al. [38] found that those participants who reported their musculoskeletal pain to be a chronic condition were more likely to experience absence OR 1.6 (95% CI 1.2, 2.32); however, none of the other studies looked at this prognostic factor.

Pain was measured by three studies Richter et al. [35], Lotters et al. (low back pain and “other” MSK pain) [34] and van Dujin et al. [38] using a 0–10-point likert scale, and all indicated that an increase in pain was significantly associated with work absence with effect sizes (OR) ranging between 1.1 and 1.3.

Steenstra et al. [41] demonstrated a dose-response effect with worsening functional ability, measured using a 0–4 scale, associated with time on absence benefits and risk of absence recurrence.

#### Summary of Findings by Workplace Systems

Work schedule was examined by Absolo et al. [39] who reported that being self-employed was protective of absence whilst having an indefinite work contract or being a “general” worker was associated with poor absence outcomes. Absolo et al. [39] also found some indication that specific work demands related to movement, e.g. frequent kneeling, flexion and rotation of the trunk were associated with absence; however, the effect sizes whilst generally significant were very small with OR between 1.05 and 1.39. Work culture was assessed by two studies both using different measures; however, both indicated that poor relationships at work and employer doubt about pain were indicators of absence [33, 41]. Richter et al. [35] found that not being satisfied at work was again associated with absence. However, the availability of modified duties during sick leave [38] and continued salary during absence [41] were also indicative of absence.

### Results: Prognostic Models

There was some overlap with studies reporting both individual prognostic factors and developing prognostic models. Overall, there were 13 prognostic models identified, some were models that had already been developed and were being tested in new populations and others were developed within a specific population.

#### Description of Included Studies: Prognostic Models

Table 2 reports the descriptive factors of the prognostic model studies. The majority of studies were undertaken using insurance health records (9 studies in total [28, 35, 37, 40, 41, 46–49]), two studies in occupational health settings [27, 45], one each in a general population [33] and primary care setting [42]. As with the prognostic factor papers, the measures of absence were varied, there was no consistency in reporting and all studies used a different outcome measure.

**Table 2:** Study characteristics (prognostic models)

Paper ID	Recruitment dates	Country	Participants	Study setting	Total number of participants	Mean age (years)	Absence definition	Absence duration (baseline)	Follow-up (years)	Predictors in final model	Prognostic model performance
Bosman et al. [45]	2016–2018	The Netherlands	On sick leave at baseline for low back pain defined using ICD codes	Occupational Health	103 Women 28 (25%)	47.4 (SD 10.49)	Still being sick listed due to low back pain at 180 days follow-up. Sick leave was defined as temporary paid leave off work with any injury or illness both work related and not work related	Mean 53.9 days (SD 30.0 days)	0.5	Catastrophising Musculoskeletal work load Disability	Calibration slope = 0.761
Branton et al. [46]	Oct 2004–May 2005	Canada	Claimants undergoing Functional Capacity Evaluation	Occupational Health	147 Women: 45 (31%)	43.3 (SD 11.1)	Future recurrence of benefits	611 days	1	Age Timeliness of referral Language barriers Attorney involvement	C statistic = 0.6 PPV 60% NPV 54%
Du Bois et al. [47]	Not reported	Belgium	Sickness fund claimants who were work in capacitated by low back pain not requiring surgery	Health records (insurance)	346 Women: 162 (47%)	41	The period an employee is absence from work with full pay as a result of injury	98% reported absence duration of > 12 weeks at baseline	0.5	Pain below the knee Not very sure to return to work within 6 months (< 10 on 10-point likert scale) Very important interference of pain in daily activities ≥ 8 on 10-point likert scale)	c statistic = 0.801: 95% Confidence Interval: 0.727–0.876 ROC correctly identified 73.7% of the non-resumers and 78.4% of the resumers
Fulton-Kehoe et al. [48]	July 2002–April 2004	USA	Workers with accepted or provisional workers compensation back sprain claims for wage replacement benefits (work disability)	Health records (insurance)	1885 Women: 603 (32%)	39.4	Long-term disability defined as the receipt of work disability payments 1 year after claim submission, where work disability payments ‘end when a worker has returned to work or has been determined to be able to work’	Mean 83 days	1	Pain interference with work Current work status (working vs not working) Radiating pain	Primary model AUC 0.79 Sensitivity 72% Specificity 78% PPV 35%
Okurowski et al. [28]	Jan 1997–Mar 2000	USA	Cases who were out of work at 3-month post-injury as the result of uncomplicated low back pain	Health records (insurance)	982 Women: 256 (26%)	Not working group 37.3 (SE0.42) Working group 35.0 (SE0.54)	Absence defined as working or not working at 6 months	3 months	0.5	Age Timeliness of referral Language barriers Attorney involvement	C statistic = 0.6 PPV 60% NPV 54%
Richter et al. [35]	Nov 2004–Dec 2006	The Netherlands	Individuals with: New work disability insurance claim episode from November 2004 until December 2006 Participants with non-specific musculoskeletal symptoms Unable to fulfil job for more than 25% according to a medical assessment	Health records (insurance)	276 Women: 20 (7%)	45 (SD 7)	Claim duration, defined as the number of calendar days the participant received work disability compensation between completion of the baseline questionnaire and one-year follow-up, without adjustment for the level of work disability (gross duration). The end of a claim period was defined as having less than 25% work disability according to a medical assessment, with a minimum duration of 4 weeks	Mixed	1	Age History of similar symptoms Duration of symptoms Self-predicted timing of RTW Job satisfaction	Not reported
Smith et al. [40]	Jan 2005–Dec 2007	Australia (Victoria)	Wage replacement claimants with an incapacity start date between January 1st, 2005 and December 31st, 2007, which were either (mental health or) back or upper extremity musculoskeletal claims; claims from full-time or part-time employees	Health records (insurance)	10,899 Women: 4197 (38.5%)	15–24y = 6.2%; 25–34y = 17.7%; 35–44y = 29.7%; 45–54y = 36.8%; 55+ = 16.9%	No. of days of total wage replacement over 2 years from first day of absence	1 day	2	Not reported	14.12% of predicted days of absence within 30 days of actual days of absence (22.31% within 31–60d; 25.68% within 61–90d; 37.89% more than 90 days out)
Steenstra et al. [41]	Jan 2005–Jun 2005	Canada	Individuals with compensated work absence after uncomplicated back injury	Health records (insurance)	1442 Women: 552 (38%)	41.3 (SD 10.5)	(1) Time on compensation benefits until RTW (2) Time to further period of compensated absence defined as recurrence of same injury	4 weeks	2	*Time on benefits:* Age Sex Physical demands Union member Early RTW Recovery expected Functional abilities Opioid prescription *Time until recurrence:* Age Sex Physical demands Opioid prescription Early RTW Functional ability	*Time on benefits:* AUC: 0.71 (95%CI0.67–0.75) at 6 months AUC: 0.79 (95%CI 0.74–0.84) at 24 months *Time until recurrence:* AUC: 0.60 (95%CI 0.54–0.64) at 1 month AUC: 0.61 (95%CI 0.57–0.65) at 3 months AUC: 0.61 (95%CI 0.57–0.65) at 6 months
Steenstra et al. [49]	Jan 2005–Jun 2005	Canada	Workers who had a lost-time claim (LTC) for an uncomplicated back injury (strain or sprain) approved by the Workplace Safety and Insurance Board (WSIB) of Ontario And participants from a RTW cohort study	Health records (insurance)	1555 Women: 605 (39%)	WSIB group 41.3 (SD 10.5) RTW group 44.0 (SD 10.2)	Time on benefits during a first claim for back pain as the length in calendar days of the first continuous episode of any wage replacement	At least 4 weeks	1	Model reported in Steenstra 2015 (above) plus: Pain score	AUC = 0.80, (95% CI 0.68, 0.91) at 180 days, AUC = 0.88, (95% CI 0.74, 1.00) at 360 days
Truchon et al. [50]	Apr 2002–Sept 2013	Canada	Compensated workers aged between 18 and 60 years, on sick leave for common LBP for a minimum of 3 weeks but no more than 11 weeks and no previous episode of LBP in the preceding year	Health records (insurance)	439 Women: 178 (40%)	38 (SD 10)	Number of days absence	Between 3 and 11 weeks Median 63 days from date of injury	1	Stress process model which included: Life events Cognitive appraisal of LBP Avoidance Coping Emotional distress Disability	The adapted stress process model explained less than 20% of the variance of number of days of absence at 6 and 12 months
Truchon et al. [37]	Not reported	Canada	French-speaking workers receiving income replacement benefits because of common LBP	Health records (insurance)	535 Women: 218 (40.7%)	42 (± 10)	Number of days absence calculated from injury date	61.7% had less than 182 days 38.3% had more than 182 days	1	Fear avoidance beliefs RTW expectations Annual family income Last level of education attained_elementary Work schedule_irregular Work concerns	C statistic = 0.73 for predicting absence > 182 days
Turner et al. [33]	Jul 2002–Jun 2003	USA	Workers 18 years or older who submitted Workers’ Compensation claims for work-related back pain and received at least 1 day of temporary total disability wage replacement (i.e. had at least 4 days of work disability, as required for receiving wage replacement)	Population based	1080 Women: 328 (31%)	45 (SD7)	The primary outcome was wage replacement compensation for temporary total disability (“work disability”) 6 months (180 days) after claim submission. Number of days of wage replacement receipt in this period was also examined (“work disability duration”) Temporary total disability payments are stopped when a worker returns to work or is judged to be medically stable and able to work	Mixed	0.5	Recovery expectations Work fear avoidance	Not reported
Westman et al. [42]	1998–2000	Sweden	Employed -18 and 65 years old, sick listed ≥ 28 days—≤ 180 days and/or had consulted the doctor about the same problem 3 times the last 12 months according to information from the referring physicians	Primary care	158 Women 110 (69%)	47 (range 24–65)	Impaired sick leave defined as a patient who maintains or increases her/his sick leave level at the follow-up or improved sick leave during follow-up defined as a patient who has decreased her/his sick leave level at follow-up	Sick leave, days previous 12 months 0–30 days 49 31 31–60 days 46 30 61–90 days 21 13 91–180 days 40 26	3	Adjusting for age and earlier sick leave (p less than 0.2) factor I (function) and factor II (pain) significantly predicted sick leave after 3 years (factors derived from Orebro) Orebro full scale	Sensitivity 63% Specificity 77% A cut-off ‘‘at-risk” score of 117 correctly classified (sensitivity) 78% of the poor outcomes (failed to reduce sick leave) and a cut-off score of 139 correctly classified 44% of those who failed to reduce their sick leave. For the same score levels, 49% and 89% of those who succeeded in reducing their sick leave were correctly classified (specificity)

##### Reporting of Models

There was wide variation in the reporting of the models included in the review (Table 3). Multivariable logistic regression was used by 3 studies [27, 28, 46] with logistic regression also reported by 3 studies [33, 42, 49] and one study reporting negative binomial regression [40]. A further 5 studies reported that Cox regression had been used for analysis [35, 37, 41, 45, 48].

**Table 3:** GRADE assessing strength of the evidence for predicting absence (prognostic factors)

			1	2	3	4	
Prognostic factor	Number of participants/studies	Effect size	QUIPS ROB	Inconsistency	Indirectness	Imprecision	Strength of evidence
Age*	28,251 participants 7 studies	Range: 0.54 -1.27 HR 0.9–0.97 OR Significant association: 5 studies all indicating older age to be protective	Downgrade 1 as more than half have high ROB	Downgrade 1 estimates of effect vary with points either side of the line of no effect and heterogeneity between studies in prognostic factor definition	No concerns	No concerns	Low
Sex^†^	7,219 participants 4 studies	Range: 0.84–1.59 HR Significant association: 4 studies mixed direction	Downgrade 1 as more than half have high ROB	Downgrade 1 estimates of effect vary with points either side of the line of no effect and heterogeneity between studies in prognostic factor definition	No concerns	No concerns	Low
Recovery expectations	3,019 participants 4 studies	Range: 0.23–2.32 HR 1.44–3,08 OR (from 1 study) Significant association: 4 studies mixed direction	Downgrade 1 as more than half have moderate/high ROB	Downgrade 1 heterogeneity between studies in prognostic factor definition	No concerns	No concerns	Low
Previous absence	7,107 participants 4 studies	Range: 0.91–1.50 HR Significant association: 2 both different directions	Downgrade 1 as more than half have high ROB	Downgrade 1 estimates of effect vary with points either side of the line of no effect and heterogeneity between studies in prognostic factor definition	No concerns	No concerns	Low
Mental health^‡^	1,691 participants 3 studies	Range: 0.83–4.64 OR Significant association: 1 study	Downgrade 1 as more than half have high ROB	Downgrade 1 estimates of effect vary with points either side of the line of no effect and heterogeneity between studies in prognostic factor definition	No concerns	No concerns	Low
Physical work demands	5,148 participants 5 studies	Range: 0.81–1.45 HR Significant association: 2 studies mixed direction	Downgrade 1 as more than half have high ROB	Downgrade 1 estimates of effect vary with points either side of the line of no effect and heterogeneity between studies in prognostic factor definition	No concerns	No concerns	Low
Work culture^§^	3,028 participants 4 studies	Range:1.11–1.85 HR Significant association: 4 studies	Downgrade 1 as more than half have moderate/high ROB	Downgrade 1 estimates of effect vary with points either side of the line of no effect and heterogeneity between studies in prognostic factor definition	No concerns	No concerns	Low
Pain^‖^	851 participants 4 studies	Range:0.96–1.17 HR Significant association: 3	Downgrade 1 as more than half have moderate/high ROB	Downgrade 1 estimates of effect vary with points either side of the line of no effect and heterogeneity between studies in prognostic factor definition	Downgrade 1 Westman et al. [42] include those who are not absent from work but do not present the results separately	No concerns	Very low
Function	2,182 participants 5 studies	Range:0.56–2.32 HR Significant association: 4 studies	Downgrade 1 as more than half have high ROB	Downgrade 1 estimates of effect vary with points either side of the line of no effect and heterogeneity between studies in prognostic factor definition	Downgrade 1 Westman et al. [42] include those who are not absent from work but do not present the results separately	No concerns	Very low
General health and quality of life	787 participants 3 studies	Range: 0.90–1.60 HR 1.51–2.26 OR Significant association: 2	Downgrade 1 as more than half have high ROB	Downgrade 1 estimates of effect vary with points either side of the line of no effect and heterogeneity between studies in prognostic factor definition	No concerns	No concerns	Low

Not able to include in the GRADE assessment due to wide heterogeneity: Other demographics; work schedule; specific work demands; work characteristics; other work variables; injury location; and other health conditions reported

*QUIPS* quality in prognostic studies, *ROB* risk of bias, *HR* hazard ratio

*2 studies do not report data to calculate effect sizes, Shiels et al. [44] and Smith et al. [40]

^†^3 studies do not report data to calculate effect sizes Shiels et al. [44], Smith et al. [40] and Truchon et al. [50]

1 study did not report data to calculate effect size Westman et al. [42]

^§^1 study did not report data to calculate effect size, Smith et al. [40]

^‖^1 study did not report data to calculate effect size, Truchon et al. [50]

Validation was carried out in only half of the included studies with 7 studies reporting that internal validation had been undertaken; however, validation was not reported in 6 studies [28, 33, 35, 40, 42, 46].

There was no consistency in the reporting of the models’ performance with most studies reporting the area under the curve or c statistic [27, 28, 37, 41, 42, 46–48] and the other studies reporting the sensitivity and specificity, [27, 37, 42, 47] positive and negative predictive value [27, 28, 47]. Five studies did not report any measure of their models’ performance [33, 35, 40, 45, 49].

None of the prognostic model papers reported the calibration of the models developed, so no observed:expected ratio or calibration slopes were presented and therefore, no assessment on the calibration of the models included here could be made.

#### Risk of Bias: Prognostic Models

**Fig. 4:**
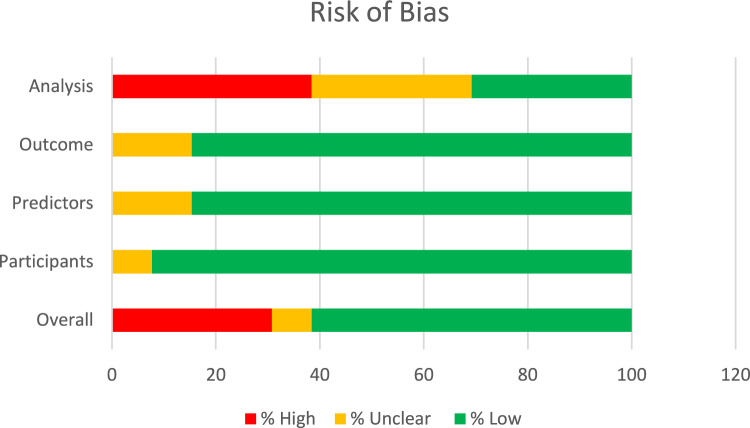
Risk of bias (PROBAST) summary judgements

Figure 4 presents the overall judgement of risk of bias based on the domains of the PROBAST tool. Overall, 62% of studies had a low risk of bias and also performed well in judgement of the domains assessing participants, predictors and outcomes. The main area for concern was the analysis where 38% of studies were at high risk of bias; this was often due to a lack of information reported in individual studies meaning assessment of how the analysis was performed was not able to be made.

#### Strength of the Evidence: Prognostic Models

Using the adapted GRADE to take account of the performance of the prognostic models included in this review, it was identified that the evidence for the use of the prognostic models was low (Table 4). This was primarily due to poor reporting of the models’ performance and one study who included a small percentage of participants who were not absent from work affecting indirectness [42]. Whilst the threshold for downgrading due to the risk of bias assessment was not met, it is worth noting that five of the 13 studies had a high or unclear risk of bias (not quite meeting the 50% required to downgrade).

**Table 4:** GRADE assessing strength of the evidence for predicting absence (models)

			1	2	3	4	
Prognostic models	Number of participants/studies	Rating of performance*	PROBAST ROB^†^	Inconsistency^‡^	Indirectness	Imprecision	Strength of evidence
	20,139/13 studies	c-statistic (AUC) Range 0.6–0.88 Sensitivity range 63–72% Specificity Range Sp. 64–78% Positive predictive value Range 35–60% Negative predictive value range 54–83%	No concerns	Downgrade 1 due to missing confidence intervals when reporting c-statistics	Downgrade 1 Westman et al. [42] include those who are not absent from work but do not present the results separately	No concerns	Low

*PROBAST* prediction model risk of bias assessment tool, *ROB* risk of bias, *AUC* area under the curve

*C-statistic or AUC > 0.7 would indicate a good/strong model

^†^Five of the 13 included studies had high or unclear risk of bias, as this did not meet the criteria for downgrading no concerns were reported here

^‡^Three studies reported confidence intervals, Dubois et al. [47] and Steenstra et al. [41, 48]

## Discussion

### Summary of Main Results

A total of 23 studies were included in this review that all reported on prognostic factors for work absence in populations with musculoskeletal pain who were absent from work. Within these 23 studies 13 had developed prognostic models aimed at predicting absence from work. A total of 110 individual prognostic factors were identified and these were grouped into those related to personal systems and workplace systems aligned with the Disability Prevention Framework, within this overarching framework groups of prognostic factors were categorised for ease of comparison, these categories included all prognostic factors measuring the same concept (Fig. 1). Overall, for both prognostic factors and prognostic models, the strength of the evidence was low to very low. This grading of the evidence is due to the heterogeneous nature of the studies where prognostic factors, outcomes and timing of outcome measurement were different across studies; furthermore, reporting of model performance was also mixed with different statistics reported or performance measures not reported at all. The Transparent Reporting of a multivariable prediction model for Individual Prognosis or Diagnosis (TRIPOD) Statement was published in 2015 [50] and so was available for just two of the papers included in the review which may account for the issues in reporting that were seen when the prognostic models were synthesised [27, 48].

### Study Strengths

We have followed the recommendations of each of the appropriate reporting checklists including Preferred Reporting Items for Systematic Reviews and Meta-Analyses (PRISMA) checklist and the CHecklist for critical Appraisal and data extraction for systematic Reviews of prediction Modelling Studies (CHARMS) [24]. Furthermore, we have followed the guidance of Damen et al. [51] who report a step-by-step guide to conducting systematic reviews of prognostic model studies, including assessment of the performance of the models.

This review has comprehensively searched the literature on prognostic factors for work absence in those with musculoskeletal conditions. By considering how to group or categorise prognostic factors a priori using the Disability Prevention Framework [13], we have ensured that sense could be made of the large number of prognostic factors identified. Being able to frame the impact of specific groups of prognostic factors within a framework mediated the impact of the heterogeneous nature of the studies, whilst we were unable to compare “like with like” we were able to assess the concepts overall and consider their contribution to predicting work absence.

The use of the GRADE system adapted to assess the strength of the evidence in prognostic factor and prognostic model studies has allowed summary judgements to be made and highlighted the inconsistencies in measurement and reporting of the studies included in the review. The adapted GRADE to include an assessment of the prognostic models’ performance has ensured that all available and pertinent data have been incorporated into the assessment of the strength of the evidence [21].

### Study Limitations

There are some limitations to the current study, principally related the heterogeneity of the studies identified as part of this review. Whilst an individual patient data meta-analysis is often considered the gold standard (Cochrane https://methods.cochrane.org/ipdma/about-ipd-meta-analyses) in assessing the influence of a factor on an outcome it is not always possible when the quality of the studies is low. Due to the heterogeneous nature of the studies included and given that studies have controlled for different potential confounders, we were unable to consider any kind of meta-analysis, nor would this be wholly appropriate for this type of review. To address this and make sense of the varied measurements and outcomes, we aimed to categorise prognostic factors a priori and as far as possible assess the contribution of each category to predicting sickness absence. It was therefore important to ensure that synthesis of findings was as structured and transparent as possible. We had planned to use a narrative synthesis [22] but felt that the Synthesis without Meta-analysis (SWiM) framework was more suitable for this review as it provides a guide with which to group, describe and report the results of systematic reviews. The SWiM framework provides a more transparent method on how the studies’ findings were synthesised allowing a clear description of the findings to be reported and a more standardised approach to be followed when considering metrics and summaries of data.

### Comparison with Other Studies

There are a number of reviews that are similar but focus on narrower populations. Kuijer et al. [52] reviewed the literature exploring the prediction of sickness absence in patients with chronic low back pain and found the same problems identified in our review with variable measurement of predictors, timing of follow-ups and differing definitions of outcome. Kuijer et al. [52] concluded that no common set of core variables could be used to predict work absence in this specific population with chronic low back pain, the current review also noted that there was no common set of core predictor variables or even outcome measures or follow-up points, indicating that little has changed since the Kuijer et al. review [52]. A recent Cochrane review by Hayden et al. [53] focussed specifically on whether recovery expectations predict outcomes including work participation for which absence is a measure, in a population with non-specific low back pain. Hayden et al. reported that there was moderate quality evidence that positive recovery expectations are strongly associated with better work participation. This finding is in part supported by the results of this review where broadly the better a participants’ recovery expectations the better the outcome. Other research has also identified previous absence as a predictor of future absences and whilst the evidence was weak in the current review the general direction of effect seen in this review supported this finding [15, 54, 55].

A recent review from Ravinskaya et al. [56] which assessed the reporting of work outcomes in randomised controlled trials also reported variability in work participation outcomes including work absence which was measured in the following ways: return to work rate, time to return to work, sick leave rate and sick leave duration. The authors concluded that a core outcome set for measurement of work participation is required and have gone on to develop that core outcome set recommending that studies including participants who are absent from work should report on the proportion of workers that return to work and time to return to work [57]. This core outcome set would ensure that comparisons between studies can better be made and may allow more pooling of data to strengthen the body of evidence.

All the studies included in this review meet the criteria for exploratory prognostic studies and models in that they are describing associations and developing prediction models as described by Kent et al. [58]. Exploratory prognostic studies are usually carried out where little is known about a condition and they are an essential early step towards a confirmatory study [58]. However, given the number of studies included in this review and the number of prognostic factors measured it is difficult to argue that little is known about what predicts work absence in those with musculoskeletal pain. Whilst there will be important predictors not measured in these studies, our review indicates that there are commonalities in the concepts that may predict work absence but there is a wide variety in how the specific prognostic factors within these concepts are measured, the main concept that indicated any predictive ability was age; however, age was measured in a variety of ways including “per year” [39], in 5-year increments [28] and in various categories [32, 35, 44] making meaningful comparisons between studies difficult. However, most prognostic studies within the field of musculoskeletal conditions are exploratory at present indicating that further research is needed to move this field forward [59]. In particular, by examining why there are differences in the extent to which models and factors predict absence.

## Conclusion

This study has systematically reviewed the evidence for prognostic factors of future sickness absence in those with musculoskeletal conditions who are currently experiencing absence. Overall, the evidence for all prognostic factors was weak, although there was some evidence that older age and better recovery expectations were protective of future absence and that previous absence was likely to predict future absences. There was weak evidence for any of the prognostic models in determining future sickness absence. Analysis was difficult due to the wide range of measures of both prognostic factors and outcome and the differing timescales for follow-up. Future research should ensure that consistent measures are employed and where possible these should be in-line with those suggested by Ravinskaya et al. [57].

## Supplementary Information

Below is the link to the electronic supplementary material.

Supplementary file1 (DOCX 34 KB)

## Data Availability

No datasets were generated or analysed during the current study.
