# Supplementary material for: Prognostic Factors and Models for Predicting Work Absence in Adults with Musculoskeletal Conditions Consulting a Healthcare Practitioner: A Systematic Review
Source: J Occup Rehabil. 2024 May 16;35(2):181–214. doi: 10.1007/s10926-024-10205-y (PMC12089206; doi:10.1007/s10926-024-10205-y)
Supplement: Supplementary file 1 — Supplementary file1 (DOCX 34 KB) [file 10926_2024_10205_MOESM1_ESM.docx]

**Search strategies**

Search strategies are as they were run in October 2020 and September 2023 with syntax specific to the database and platform searched. The Medline and EMBASE search strategies incorporated several search filters for identifying prognosis studies as part of a methodological project to assess their relative value for this type of systematic review.

The search filters (& references) used were:

**Medline filters**

**Buckingham** - Parker R, Tougas ME, Hayden JA. Validating prognosis search filters using relative recall based on prognosis systematic reviews. Poster presented at: 21st Cochrane Colloquium; Quebec City, QC, Canada; 19–23 Sep 2013. Conference poster from the Author.

**Haynes broad search** –

Keogh C, Wallace E, O'Brien KK, Murphy PJ, Teljeur C, McGrath B, Smith SM, Doherty N, Dimitrov BD, Fahey T. Optimized retrieval of primary care clinical prediction rules from MEDLINE to establish aWeb-based register. J Clin Epi. 2011;64(8):848-60.

Geersing GJ, Bouwmeester W, Zuithoff P, Spijker R, Leeflang M, Moons K. Search filters for finding prognostic and diagnostic prediction studies in Medline to enhance systematic reviews. PLoS One. 2012;7(2):e32844

**Hedges best accuracy 1986**/ **Hedges best sensitivity 1986 search/ Hedges best sensitivity 1991 search** - Haynes RB, Wilczynski N, McKibbon KA, Walker CJ, Sinclair JC. Developing optimal search strategies for detecting clinically sound studies in MEDLINE. Journal of the American Medical Informatics Association 1994;1(6):447‐58

**Hedges best sensitivity with specificity >50%** - Wilczynski NL, Haynes RB. Developing optimal search strategies for detecting clinically sound prognostic studies in MEDLINE: an analytic survey. BMC Med. 2004;2:23

**Hedges plus ‘natural history’ (Parker et al.) & Parker's Inclusive search** –

Parker R, Tougas ME, Hayden JA. Validating prognosis search filters using relative recall based on prognosis systematic reviews. Poster presented at: 21st Cochrane Colloquium; Quebec City, QC, Canada; 19–23 Sep 2013

Boulos L, Ogilvie R, Hayden J A. Search methods for prognostic factor systematic reviews: a methodologic investigation. J Med Lib Assoc [Online]. 2021; 109(1): 23–32.

**Yale best terms search & Yale (Translated to Ovid from Pubmed)** –

Reported on Yale web site https://library.medicine.yale.edu/tutorials/577 [Last accessed: 14 Dec 2023]

Kok R, Verbeek JAHM, Faber B, van Dijk, Frank JH, Hoving, Jan L A search strategy to identify studies on the prognosis of work disability: a diagnostic test framework. BMJ Open. 2015;5:e006315. doi:10.1136/bmjopen-2014-006315

**EMBASE filters**

**Hedges Single Term Best Sensitivity plus Spec 50percent_EMBASE/ Hedges Best Optimised_EMBASE/ Hedges Single Term Best Sensitivity plus Spec 50percent_EMBASE** –

Holland JL, Wilczynski NL, Haynes RB. Optimal search strategies for identifying sound clinical prediction studies in EMBASE. BMC Med Inform Dec Mak. 2005;5:11

Wilczynski NL, Haynes RB. Optimal search strategies for detecting clinically sound prognostic studies in EMBASE: an analytic survey. J Am Med Inform Assoc 2005;12(4):481-5.

**Ovid MEDLINE(R) and Epub Ahead of Print, In-Process & Other Non-Indexed Citations, Daily and Versions(R) <1946 to October 06, 2020>**

1. exp Pain/

2. pain.ti,ab.

3. 1 or 2

4. joint$.mp.

5. exp Back/ or back.mp.

6. exp Knee/ or knee.mp.

7. shoulder.mp. or exp Shoulder/

8. exp Neck/ or neck.mp.

9. exp Elbow/ or elbow.mp.

10. exp Hand/ or hand$.mp.

11. exp Hip/ or hip.mp.

12. exp Foot/ or foot.mp.

13. feet$.mp.

14. or/4-13

15. 3 and 14

16. exp musculoskeletal diseases/

17. (musculoskeletal adj2 (disease$ or disorder$ or pain)).ti,ab.

18. (chronic adj2 pain).ti,ab.

19. (osteoarthr$ or arthriti$ or arthrosis or arthroses or arthrotic).ti,ab.

20. (gout$ or "polymyalgia rheumatica").ti,ab.

21. fibromyalgia.ti,ab.

22. or/15-21

23. Return to Work/

24. (work adj2 absen$).mp.

25. (return$ adj2 work$).mp.

26. (sick$ adj2 (leave or absen$ or certif$ or note$ or pay$ or paid)).mp.

27. (work$ adj2 participation).mp.

28. (fit$ adj2 note$).mp.

29. Sick Leave/

30. Absenteeism/

31. absenteeism.mp.

32. incapacity.mp.

33. (occupation$ adj2 (leave or absen$ or return$)).mp.

34. ((work or occupation$ or job) adj2 (ability or able or disab$)).mp.

35. 23 or 24 or 25 or 26 or 27 or 28 or 29 or 30 or 31 or 32 or 33 or 34

36. 22 and 35

[**Prognosis filter 1 – Buckingham**]

37. disease progression/

38. prognos$.mp.

39. predict$.mp.

40. outcome$.mp.

41. follow-up.mp.

42. natural progress$.mp.

43. natural course$.mp.

44. natural history.mp.

45. disease progress$.mp.

46. disease course$.mp.

47. disease history.mp.

48. 37 or 38 or 39 or 40 or 41 or 42 or 43 or 44 or 45 or 46 or 47

49. cohort studies/

50. cohort$.mp.

51. compar$.mp.

52. longitudinal.mp.

53. prospective$.mp.

54. multi-variate.mp.

55. reproducib$.mp.

56. 49 or 50 or 51 or 52 or 53 or 54 or 55

57. 48 and 56

[**Prognosis filter 2 – Haynes broad search**]

58. exp predictive value of tests/

59. exp observer variation/

60. predict$.ti,ab.

61. scor$.ti,ab.

62. observ$.ti,ab.

63. 58 or 59 or 60 or 61 or 62

[**Prognosis filter 3 – Hedges best accuracy 1986]**

64. prognosis/

65. follow up studies/

66. mortality.fs.

67. course.tw.

68. (prognostic and factor$).tw.

69. (natural and history).tw.

70. 64 or 65 or 66 or 67 or 68 or 69

[**Prognosis filter 4 – Hedges best sensitivity 1986 search]**

71. exp cohort studies/

72. prognosis/

73. mortality.fs.

74. predict$.tw.

75. course.tw.

76. (natural and history).tw.

77. 71 or 72 or 73 or 74 or 75 or 76

[**Prognosis filter 5 – Hedges best sensitivity 1991 search]**

78. exp mortality/

79. Follow-Up Studies/

80. incidence/

81. mortality.fs.

82. prognos$.tw.

83. predict$.tw.

84. course$.tw.

85. 78 or 79 or 80 or 81 or 82 or 83 or 84

[**Prognosis filter 6 – Hedges best sensitivity with specificity >50%]**

86. exp mortality/

87. incidence/

88. follow-up studies/

89. prognos$.tw.

90. predict$.tw.

91. course$.tw.

92. 86 or 87 or 88 or 89 or 90 or 91

[**Prognosis filter 7 – Hedges plus ‘natural history’ (Parker et al.)]**

93. incidence/

94. follow-up studies/

95. exp mortality/

96. prognos$.mp.

97. predict$.mp.

98. course$.mp.

99. cohort.ti,ab.

100. (first and episode).ti,ab.

101. "natural history".mp.

102. 93 or 94 or 95 or 96 or 97 or 98 or 99 or 100

[**Prognosis filter 8 – Parker's Inclusive search]**

103. prognos$.af.

104. predict$.af.

105. course.af.

106. (incidence or epidemiology).af.

107. mortality.af.

108. cohort.af.

109. "follow-up study".af.

110. "follow-up studies".af.

111. "natural history".ti,ab.

112. 103 or 104 or 105 or 106 or 107 or 108 or 109 or 110 or 111

[**Prognosis filter 9 – Yale best terms search]**

113. exp prognosis/

114. exp cohort studies/

115. exp disease progression/

116. 113 or 114 or 115

**[Prognosis filter 10 – Yale (Translated from Pubmed)]**

117. exp prognosis/

118. exp cohort studies/

119. exp mortality/

120. exp morbidity/

121. exp "Outcome Assessment (Health Care)"/

122. exp disease progression/

123. exp survival analysis/

124. "natural history".af.

125. inception cohort*.af.

126. prognost$.ti,ab.

127. predict$.ti,ab.

128. course.ti,ab.

129. outcome$.ti,ab.

130. 117 or 118 or 119 or 120 or 121 or 122 or 123 or 124 or 125 or 126 or 127 or 128 or 129

131. 36 and 57

132. 36 and 63

133. 36 and 70

134. 36 and 77

135. 36 and 85

136. 36 and 92

137. 36 and 102

138. 36 and 112

139. 36 and 116

140. 36 and 130

141. 131 or 132 or 133 or 134 or 135 or 136 or 137 or 138 or 139 or 140

**EMBASE (OVID) Search strategy – 06/10/2020**

1. exp pain/

2. pain.ti,ab.

3. 1 or 2

4. joint$.mp.

5. exp back/ or back.mp.

6. exp knee/ or knee$.mp.

7. exp shoulder/ or shoulder.mp.

8. exp neck/ or neck.mp.

9. exp elbow/ or elbow.mp.

10. exp hand/ or hand$.mp.

11. exp hip/ or hip.mp.

12. exp foot/ or (foot or feet).mp.

13. 4 or 5 or 6 or 7 or 8 or 9 or 10 or 11 or 12

14. 3 and 13

15. exp musculoskeletal pain/

16. exp osteoarthritis/

17. (musculoskeletal adj2 (disease$ or disorder$ or pain)).ti,ab.

18. (chronic adj2 pain).ti,ab.

19. (osteoarthr$ or arthriti$ or arthrosis or arthoses or arthrotic).ti,ab.

20. (gout$ or "polymyalgia rheumatica").ti,ab.

21. fibromyalgia.ti,ab.

22. 14 or 15 or 16 or 17 or 18 or 19 or 20 or 21

23. absenteeism/ or return to work/

24. (work adj2 absen$).mp.

25. (return$ adj2 work$).mp.

26. (sick$ adj2 (leave or absen$ or certif$ or note$ or pay$ or paid or benefit$)).mp.

27. (work$ adj2 participat$).mp.

28. (fit$ adj2 note$).mp.

29. exp medical leave/

30. absenteeism.mp.

31. incapacity.mp.

32. (occupation$ adj2 (leave or absen$ or return$)).mp.

33. ((work or occupation$ or job) adj2 (ability or able or disab$)).mp.

34. 23 or 24 or 25 or 26 or 27 or 28 or 29 or 30 or 31 or 32 or 33

35. 22 and 34

36. limit 35 to embase

**[Prognosis Filter_Hedges Best Sensitivity plus Spec 50percent_EMBASE]**

37. exp disease course/

38. risk$.mp.

39. diagnos$.mp.

40. follow-up.mp.

41. ep.fs.

42. outcome.tw.

43. or/37-42

**[Prognosis Filter_Hedges Best Optimised_EMBASE]**

44. follow-up.mp.

45. prognos$.tw.

46. ep.fs.

47. or/44-46

**[Prognosis Filter_Hedges Single Term Best Sensitivity plus Spec 50percent_EMBASE]**

48. exp "general aspects of disease"/

49. 36 and 43

50. 36 and 47

51. 36 and 48

52. 49 or 50 or 51

**CINAHL (EBSCO) – 06/10/2020**

S35 S26 AND S34

S34 S27 OR S28 OR S29 OR S30 OR S31 OR S32 OR S33

S33 TI ( cohort or prospective* or longitudinal ) OR AB ( cohort or prospective* or longitudinal )

S32 TI ( (disease* or natural or clinical) N2 (history or course or progress*) ) OR AB ( (disease* or natural or clinical) N2 (history or course or progress*) )

S31 (MH "Disease Progression+")

S30 TI ( prognos* or predict* ) OR AB ( prognos* or predict* )

S29 (MH "Epidemiological Research")

S28 (MH "Epidemiology")

S27 (MH "Prognosis")

S26 S13 AND S25

S25 S14 OR S15 OR S16 OR S17 OR S18 OR S19 OR S20 OR S21 OR S22 OR S23 OR S24

S24 TI ( ((work* or occupation* or job) N2 (ability or able or disab*)) ) OR AB ( ((work* or occupation* or job) N2 (ability or able or disab*)) )

S23 TI ( (occupation* N2 (leave or absen* or return*)) ) OR AB ( (occupation* N2 (leave or absen* or return*)) )

S22 TI incapacity OR AB incapacity

S21 TI absenteeism OR AB absenteeism

S20 TI fit* N2 note* OR AB fit* N2 note*

S19 TI ( (sick* N2 (leave or absen* or certif* or note* or pay* or paid)) ) OR AB ( (sick* N2 (leave or absen* or certif* or note* or pay* or paid)) )

S18 TI (return* N2 work*) OR AB (return* N2 work*)

S17 TI (work N2 absen* or participat*) OR AB (work N2 absen* or participat*)

S16 (MH "Absenteeism")

S15 (MH "Sick Leave")

S14 (MH "Job Re-Entry")

S13 S7 OR S8 OR S9 OR S10 OR S11 OR S12

S12 TI ( (gout* or "polymyalgia rheumatica" or fibromyalgia) ) OR AB ( (gout* or "polymyalgia rheumatica" or fibromyalgia) )

S11 TI ( (osteoarthr* or arthriti* or arthrosis or arthroses or arthrotic) ) OR AB ( (osteoarthr* or arthriti* or arthrosis or arthroses or arthrotic) )

S10 TI (chronic N2 pain*) OR AB (chronic N2 pain*)

S9 TI ( (musculoskeletal N2 (disease* or disorder*)) ) OR AB ( (musculoskeletal N2 (disease* or disorder*)) )

S8 (MH "Musculoskeletal Diseases+")

S7 S3 AND S6

S6 S4 OR S5

S5 TI (musculoskeletal or joint* or back or knee* or shoulder* or neck or elbow* or hand* or hip or foot or finger* or thumb* or foot or feet ) OR AB (musculoskeletal or joint* or back or knee* or shoulder* or neck or elbow* or hand* or hip or foot or finger* or thumb* or foot or feet )

S4 (MH "Joints+")

S3 S1 OR S2

S2 TI pain OR AB pain

S1 (MH "Pain+")

**PsycINFO (EBSCO) – 06/10/2020**

**S19** S7 AND S18

**S18** S8 OR S9 OR S10 OR S11 OR S12 OR S13 OR S14 OR S15 OR S16 OR S17

**S17** TI ( ((work OR occupation* OR job) N2 (ability OR able OR disab*)) ) OR AB ( ((work OR occupation* OR job) N2 (ability OR able OR disab*)) )

**S16** TI ( (occupation* N2 (leave OR absen* OR return*)) ) OR AB ( (occupation* N2 (leave OR absen* OR return*)) )

**S15** DE "Reemployment"

**S14** TI fit* N2 note* OR AB fit* N2 note*

**S13** TI work* N2 participation OR AB work* N2 participation

**S12** TI ( (sick* N2 (leave OR absen* OR certif* OR note* OR pay* OR paid)) ) OR AB ( (sick* N2 (leave OR absen* OR certif* OR note* OR pay* OR paid)) )

**S11** TI work* N2 return* OR AB work* N2 return*

**S10** TI work* N2 absen* OR AB work* N2 absen*

**S9** TI absenteeism OR AB absenteeism

**S8** DE "Employee Absenteeism"

**S7** S1 OR S2 OR S3 OR S4 OR S5 OR S6

**S6** TI ( ((knee OR hip OR back OR neck OR shoulder) N2 pain*) ) OR AB ( ((knee OR hip OR back OR neck OR shoulder) N2 pain*) )

**S5** TI ( (gout* OR "polymyalgia rheumatica" OR fibromyalgia) ) OR AB ( (gout* OR "polymyalgia rheumatica" OR fibromyalgia) )

**S4** TI ( (osteoarthr* OR arthriti* OR arthrosis OR arthroses OR arthrotic) ) OR AB ( (osteoarthr* OR arthriti* OR arthrosis OR arthroses OR arthrotic) )

**S3** TI (chronic N2 pain*) OR AB (chronic N2 pain*)

**S2** TI ( ((musculoskeletal OR joint OR joints) N2 (pain* OR disease OR disorder)) ) OR AB ( ((musculoskeletal OR joint OR joints) N2 (pain* OR disease OR disorder)) )

**S1** DE "Musculoskeletal Disorders" OR DE "Bone Disorders" OR DE "Bruxism" OR DE "Joint Disorders" OR DE "Muscular Disorders"

**Emcare (OVID) – 06/10/2020**

1. exp pain/

2. pain.ti,ab.

3. 1 or 2

4. joint$.mp.

5. exp back/ or back.mp.

6. exp knee/ or knee$.mp.

7. exp shoulder/ or shoulder.mp.

8. exp neck/ or neck.mp.

9. exp elbow/ or elbow.mp.

10. exp hand/ or hand$.mp.

11. exp hip/ or hip.mp.

12. exp foot/ or (foot or feet).mp.

13. 4 or 5 or 6 or 7 or 8 or 9 or 10 or 11 or 12

14. 3 and 13

15. exp musculoskeletal pain/

16. exp osteoarthritis/

17. (musculoskeletal adj2 (disease$ or disorder$ or pain)).ti,ab.

18. (chronic adj2 pain).ti,ab.

19. (osteoarthr$ or arthriti$ or arthrosis or arthoses or arthrotic).ti,ab.

20. (gout$ or "polymyalgia rheumatica").ti,ab.

21. fibromyalgia.ti,ab.

22. 14 or 15 or 16 or 17 or 18 or 19 or 20 or 21

23. absenteeism/ or return to work/

24. (work adj2 absen$).mp.

25. (return$ adj2 work$).mp.

26. (sick$ adj2 (leave or absen$ or certif$ or note$ or pay$ or paid or benefit$)).mp.

27. (work$ adj2 participat$).mp.

28. (fit$ adj2 note$).mp.

29. exp medical leave/

30. absenteeism.mp.

31. incapacity.mp.

32. (occupation$ adj2 (leave or absen$ or return$)).mp.

33. ((work or occupation$ or job) adj2 (ability or able or disab$)).mp.

34. 23 or 24 or 25 or 26 or 27 or 28 or 29 or 30 or 31 or 32 or 33

35. 22 and 34

36. prognosis/ or disease course/

37. exp epidemiology/

38. (prognos* or predict*).ti,ab,kw.

39. ((disease$ or natural or clinical) adj2 (history or course or progress$)).ti,ab,kw.

40. (cohort or prospective$ or longitudinal).ti,ab,kw.

41. 36 or 37 or 38 or 39 or 40

42. 35 and 41

**HMIC (OVID) – 06/10/2020**

1. exp pain/

2. pain.ti,ab.

3. 1 or 2

4. joint$.mp.

5. exp back/ or back.mp.

6. exp Knees/ or knee$.mp.

7. exp shoulder/ or shoulder.mp.

8. exp neck/ or neck.mp.

9. exp Elbows/ or elbow.mp.

10. exp Hands/ or hand$.mp.

11. exp hip/ or hip.mp.

12. exp Feet/ or (foot or feet).mp.

13. 4 or 5 or 6 or 7 or 8 or 9 or 10 or 11 or 12

14. 3 and 13

15. exp musculoskeletal system diseases/

16. (musculoskeletal adj2 (disease$ or disorder$ or pain)).ti,ab.

17. (chronic adj2 pain).ti,ab.

18. (osteoarthr$ or arthriti$ or arthrosis or arthoses or arthrotic).ti,ab.

19. (gout$ or "polymyalgia rheumatica").ti,ab.

20. fibromyalgia.ti,ab.

21. 14 or 15 or 16 or 17 or 18 or 19 or 20

22. absenteeism/

23. (work adj2 absen$).mp.

24. (return$ adj2 work$).mp.

25. (sick$ adj2 (leave or absen$ or certif$ or note$ or pay$ or paid or benefit$)).mp.

26. (work$ adj2 participat$).mp.

27. (fit$ adj2 note$).mp.

28. absenteeism.mp.

29. incapacity.mp.

30. (occupation$ adj2 (leave or absen$ or return$)).mp.

31. ((work or occupation$ or job) adj2 (ability or able or disab$)).mp.

32. 22 or 23 or 24 or 25 or 26 or 27 or 28 or 29 or 30 or 31

33. 21 and 32

**AMED (OVID) – 06/10/2020**

1. exp pain/

2. pain.ti,ab.

3. 1 or 2

4. joint$.mp.

5. exp back/ or back.mp.

6. exp knee/ or knee$.mp.

7. exp shoulder/ or shoulder.mp.

8. exp neck/ or neck.mp.

9. exp elbow/ or elbow.mp.

10. exp hand/ or hand$.mp.

11. exp hip/ or hip.mp.

12. exp foot/ or (foot or feet).mp.

13. 4 or 5 or 6 or 7 or 8 or 9 or 10 or 11 or 12

14. 3 and 13

15. exp musculoskeletal pain/

16. exp musculoskeletal disease/

17. (musculoskeletal adj2 (disease$ or disorder$ or pain)).ti,ab.

18. (chronic adj2 pain).ti,ab.

19. (osteoarthr$ or arthriti$ or arthrosis or arthoses or arthrotic).ti,ab.

20. (gout$ or "polymyalgia rheumatica").ti,ab.

21. fibromyalgia.ti,ab.

22. 14 or 15 or 16 or 17 or 18 or 19 or 20 or 21

23. absenteeism/

24. (work adj2 absen$).mp.

25. (return$ adj2 work$).mp.

26. (sick$ adj2 (leave or absen$ or certif$ or note$ or pay$ or paid or benefit$)).mp.

27. (work$ adj2 participat$).mp.

28. (fit$ adj2 note$).mp.

29. absenteeism.mp.

30. incapacity.mp.

31. (occupation$ adj2 (leave or absen$ or return$)).mp.

32. ((work or occupation$ or job) adj2 (ability or able or disab$)).mp.

33. 23 or 24 or 25 or 26 or 27 or 28 or 29 or 30 or 31 or 32

34. 22 and 33

**Web of Science Core Content 1970-06/10/2020**

# 15 #14 AND #13

# 14 TOPIC: ((prognos* or predict* or cohort or course or “follow up” or “natural history” or epidemiol*) )

# 13 #12 AND #7

# 12 #11 OR #10 OR #9 OR #8

# 11 TOPIC: (absenteeism)

# 10 TOPIC: ((sick or sickness) NEAR/2 (absence or leave or certificate* or note) )

# 9 TOPIC: ("work absence")

# 8 TOPIC: ("return to work")

# 7 #6 OR #5 OR #4 OR #3 OR #2 OR #1

# 6 TOPIC: (osteoarthritis)

# 5 TOPIC: (neck pain)

# 4 TOPIC: (shoulder pain)

# 3 TOPIC: (back pain)

# 2 TOPIC: (joint pain)

# 1 TOPIC: (musculoskeletal)
